# Causes of discordance between noninvasive prenatal testing and fetal chromosomal status: a 10-year cohort study of 97, 743 cases

**DOI:** 10.3389/fendo.2026.1945762

**Published:** 2026-10-20

**Authors:** Haimei Qi, Haijun Chen, Xianping Yuan, Jinhui Gan, Huifeng Liu, Xinxing Xie, Qing Xie, Junkun Chen, Shichun Shen, Liyun Song

**Affiliations:** 1 Department of Clinical Laboratory, Ganzhou Women and Children’s Health Care Hospital, Ganzhou, China; 2 Department of Medical Genetics, Ganzhou Women and Children’s Health Care Hospital, Ganzhou, China; 3 Department of Obstetrics, Ganzhou Women and Children’s Health Care Hospital, Ganzhou, China; 4 Department of Clinical Laboratory, Ganzhou People’s Hospital, Ganzhou, China

**Keywords:** chromosomal aneuploidy, confined placental mosaicism, false negative, false positive, noninvasive prenatal testing, vanishing twin, Z-score

## Abstract

**Background:**

Noninvasive prenatal testing (NIPT) has been widely adopted for screening common fetal chromosomal aneuploidies, yet discordance between NIPT results and true fetal karyotypes remains a critical challenge limiting its clinical utility.

**Methods:**

We retrospectively analyzed 97, 743 pregnant women who underwent NIPT at Ganzhou Women and Children’s Health Care Hospital between April 2015 and December 2024 to evaluate the performance of NIPT and investigate the causes of discordance between NIPT results and fetal outcomes.

**Results:**

A total of 481 NIPT-positive cases were identified, yielding an overall positive rate of 0.49%. Of these, 380 cases underwent confirmatory testing (377 via invasive prenatal diagnosis and 3 via postnatal karyotyping). The overall positive predictive value (PPV) for the three common trisomies was 71.84%, with T21 showing the highest PPV (84.38%), followed by T18 (56.00%) and T13 (4.76%). Stratified analysis by Z-score demonstrated that PPVs for both T21 and T18 increased progressively with higher Z-score. For T21, PPV rose from 21.95% (Z: 3-6) to 100% (Z > 20); a similar upward trend was observed for T18. False-positive results were attributed to multiple mechanisms, including maternal chromosomal abnormalities (4 full 47, XX, + 21, 1 mosaic), vanishing twin (7 cases), uniparental disomy (2 cases), and confined placental mosaicism (CPM) (2 cases). Only 3 false-negative cases were identified, predominantly due to low-level placental mosaicism.

**Conclusions:**

Discordance between NIPT and fetal karyotype exhibits a clear directional divergence: false-positive results are etiologically heterogeneous, involving maternal chromosomal abnormalities, substantially underestimated vanishing twin, CPM, and technical factors, whereas false-negative results are predominantly attributable to low-level CPM. Systematic analysis of Z-scores, maternal characteristics, and follow-up data can effectively identify most discordance causes, providing precise evidence for genetic counseling and pregnancy management. Our analytical framework provides a practical reference for optimizing NIPT screening strategies and reducing unnecessary invasive procedures in other regions.

## 1 Introduction

Chromosomal aneuploidy refers to a deviation in the number of one or more chromosomes from the normal diploid state, typically resulting from nondisjunction events during meiosis or mitosis (1), and advanced maternal age significantly increases the risk of fetal chromosomal aneuploidy (2). The most common clinically observed chromosomal aneuploidies include Down syndrome (Trisomy 21, T21), Edwards syndrome (Trisomy 18, T18), and Patau syndrome (Trisomy 13, T13) (3–5). These genetic abnormalities represent major causes of birth defects, spontaneous abortion, and genetic disorders (6). Consequently, the detection of chromosomal aneuploidy during pregnancy has become a priority for birth defect prevention. However, definitive diagnostic procedures such as chorionic villus sampling and amniocentesis are not only costly but also carry an inherent risk of miscarriage, underscoring the urgent need for a cost-effective and safe detection method (7).

The placenta, acting as an endocrine organ during pregnancy, secretes a variety of hormones (8). In cases of fetal chromosomal aneuploidy, the hormonal profile secreted by the placenta exhibits distinct characteristics compared with that of a normal pregnancy (9). Based on this observation, serum screening measuring placenta-derived hormones from maternal peripheral blood was developed. Nevertheless, serum screening has limitations, including a low positive predictive value (PPV) and a considerable risk of false-negative results (9). In 1997, Lo et al. identified cell-free fetal DNA (cffDNA) in maternal peripheral blood (10), and subsequent studies confirmed that this cffDNA primarily originates from apoptotic placental cells released into the maternal circulation. Since the placenta and the fetus derive from the same fertilized egg, they theoretically share identical genetic material. In recent years, noninvasive prenatal testing (NIPT), which detects fetal chromosomal aneuploidies based on cffDNA, has been widely adopted for screening common fetal chromosomal abnormalities and is gradually replacing serum screening (11). Numerous studies have demonstrated that NIPT exhibits high sensitivity and specificity for detecting T21, T18, and T13 (12). However, due to technical and biological limitations, NIPT still suffers from issues such as false positives, false negatives, and a suboptimal PPV, leading to occasional discordance between screening results and the true fetal karyotype. This not only causes anxiety for pregnant women but also poses challenges for clinical counseling and decision-making.

This study collected clinical data from 97, 743 pregnant women who underwent NIPT at our hospital between April 2015 and December 2024. We systematically analyzed the prenatal diagnostic results of 481 NIPT-positive cases and the follow-up data of negative cases. The aim was to thoroughly investigate the causes of discordance between NIPT results and the true fetal karyotype, evaluate the clinical value of NIPT in screening for fetal chromosomal abnormalities, and thereby provide evidence-based guidance for optimizing screening strategies and genetic counseling.

## 2 Materials and methods

### 2.1 Study design and participants

This retrospective study included 97, 743 pregnant women who underwent NIPT at Ganzhou Women and Children’s Health Care Hospital between April 2015 and December 2024. All participants had valid NIPT results and complete demographic and clinical data. Prior to testing, all participants were informed of the testing methodology, the spectrum of diseases covered, technical limitations, and associated risks, and written informed consent was obtained from each participant. This study was approved by the Ethics Committee of Ganzhou Women and Children’s Health Care Hospital (approval number: 2024056) and was conducted in accordance with the Declaration of Helsinki.

### 2.2 NIPT procedures

Peripheral venous blood samples (5–10 mL) were collected into EDTA-anticoagulated tubes or cell-free nucleic acid preservation tubes. Plasma was separated by two-step centrifugation at 1, 600 × g (4 °C) for 10 min, followed by 16, 000 × g (4 °C) for 10 min. Plasma was used to extract cffDNA using the QIAamp DSP DNA Blood Mini Kit (Qiagen, Hilden, Germany). Extracted DNA underwent end-repair using the Ion Plus Fragment Library Kit (Life Technologies, Carlsbad, CA, USA), followed by magnetic bead-based size selection (<230 bp) to enrich cffDNA fragments. The size-selected DNA was ligated to adapters and amplified by PCR. Library quantification was performed using quantitative PCR, and concentrations were normalized by dilution to ensure uniform sequencing density. Pooled libraries were sequenced on the Bioelectron-seq 4000 platform (CapitalBio, Beijing, China).

Sequencing data were analyzed using NIPT Data Analysis Management Software (CapitalBio Genomics, Beijing, China). Raw data were filtered by the following criteria: mean read length >100 bp, sequencing quality score (Q20) >50%, GC content 38%-45%, and cffDNA fraction ≥4%. Clean reads were aligned to the human reference genome (hg19). After alignment, low-quality alignments and PCR duplicates were removed to obtain unique reads, with a minimum of 3.5 million unique reads per sample. Chromosomes were segmented into 20-kb bins, and the percentage of unique reads relative to autosomal reads was calculated. Chromosomal aneuploidies were determined by |Z-score| ≥ 3, based on statistical deviation analysis. Further methodological details have been described previously (13, 14).

### 2.3 Prenatal diagnosis

All women with NIPT-positive results received genetic counseling. Upon written informed consent, amniocentesis was performed at 16–22 weeks of gestation to collect 20–30 mL of amniotic fluid. Amniotic fluid samples were cultured for conventional G-banding karyotyping at a resolution of 400 bands, or were directly analyzed by chromosomal microarray analysis (CMA) using the CytoScan™ 750K Array (Affymetrix, Santa Clara, CA, USA). CMA results were analyzed using the ChAS software (Affymetrix). For cases with negative NIPT results but abnormal ultrasound findings, prenatal diagnosis was also offered and performed following the same protocol.

### 2.4 Placental tissue validation

Placental tissues were obtained from women with false-positive or false-negative NIPT results after delivery or induced abortion, with written informed consent. Genomic DNA was extracted from placental samples using the QIAamp DSP DNA Blood Mini Kit (Qiagen). DNA libraries were prepared using PCR amplification and subjected to circularization and DNA nanoball preparation. Sequencing was performed on the MGISEQ-2000 platform (MGI, Shenzhen, China) with a minimum of 5 million reads per sample. Raw sequencing reads were aligned to the human reference genome (hg19), and chromosomal abnormalities were identified using Halos software (MGI).

### 2.5 Pregnancy and neonatal follow-up

For NIPT-positive cases who declined prenatal diagnosis, pregnancy outcomes were documented through telephone follow-up at 6 months after the estimated date of delivery. For NIPT-negative cases, routine prenatal examinations were recommended, and follow-up was conducted by telephone at 6 months postpartum in accordance with national guidelines. The primary objective was to confirm the presence or absence of trisomy 21, trisomy 18, or trisomy 13, and to record fetal sex. If birth defects were reported, further karyotyping or genetic testing was recommended for definitive diagnosis.

### 2.6 Mechanistic investigation of false-positive cases

For false-positive cases, additional mechanistic investigations were performed when at least one of the following was available: maternal peripheral blood karyotyping, placental tissue verification, or sex-discordance analysis through postnatal follow-up. Maternal karyotyping was performed only when clinical features suggestive of maternal chromosomal abnormality were present, such as intellectual disability. Of the 107 false-positive cases, placental tissue was obtained for verification in only 6, because most patients declined or samples could not be collected. Sex-discordance analysis was performed by comparing the fetal sex predicted from raw NIPT data with the postnatal sex; vanishing twin was considered when sex discordance was present or when a history of twin demise was reported. Cases without any of these additional data were not included in the mechanistic analysis. The reasons for missing data included absence of clinical symptoms prompting maternal karyotyping, patient refusal or unavailability of placental tissue, and undetectable vanishing twin when the demised fetus had the same sex as the surviving fetus or when the demise was not reported.

### 2.7 Statistical analysis

All statistical analyses were performed using SPSS version 27.0 (IBM Corp., Armonk, NY, USA). Continuous variables were presented as mean ± standard deviation ( $\overset{-}{x}$ ± s), and categorical variables as rates (%). Comparisons of continuous variables between two groups were performed using independent samples t-tests. Categorical variables were compared using the chi-square (χ²) test; when expected frequencies in any cell were less than 5, Fisher’s exact test was used. A P value< 0.05 was considered statistically significant. All statistical tests were two-sided.

## 3 Results

### 3.1 Verification results and pregnancy outcomes of NIPT-positive cases

Among the 97, 743 pregnant women who underwent NIPT, a total of 481 positive cases were identified (**Supplementary Table S1**), yielding an overall positive rate of 0.49%. Specifically, 348 cases were positive for T21 (positive rate 0.36%), 74 for T18 (0.08%), and 59 for T13 (0.06%) (**Table 1**). Of the 481 positive cases, 377 underwent invasive prenatal diagnosis, with a diagnostic uptake rate of 78.38% (**Figure 1**).

**Table 1:** Verification results and pregnancy outcomes of NIPT-positive cases.

Variable	T21	T18	T13	Overall
NIPT-positive cases (positive rate)	348 (0.36%)	74 (0.08%)	59 (0.06%)	481 (0.49%)
Invasive prenatal diagnosis (uptake rate)	285 (81.90%)	50 (67.57%)	42 (71.19%)	377 (78.38%)
True-positive cases by prenatal diagnosis	240	28	2	270
Corrected true-positive cases^a^	243	28	2	273
Corrected PPV	84.38%	56.00%	4.76%	71.84%
Termination of pregnancy	232	28	2	262
Continued pregnancy/Selective reduction with live birth^b^	8^b^	0	0	8

^a^Three additional T21 true-positive cases confirmed by postnatal peripheral blood karyotyping were included in the corrected PPV analysis. ^b^Includes 4 cases of continued pregnancy with T21 and 4 twin pregnancies that underwent selective fetal reduction and delivered normal infants.

**Figure 1:**
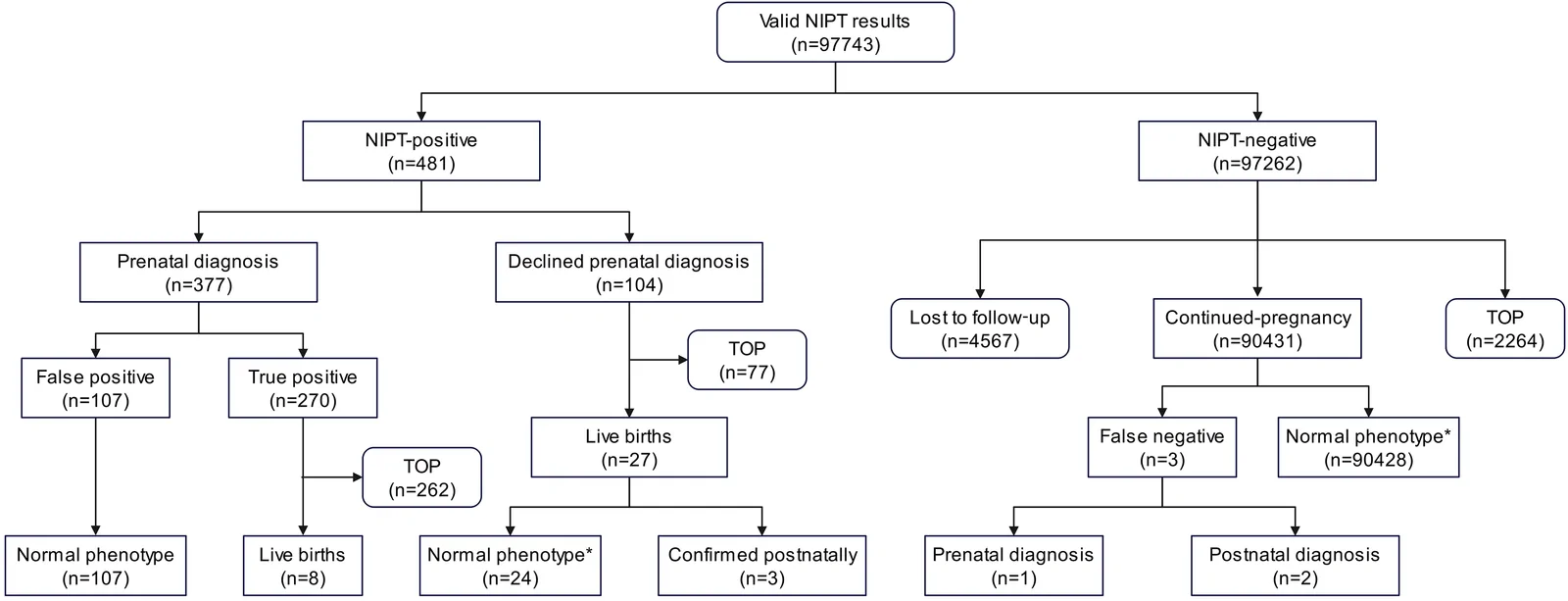
Flowchart of the study population and pregnancy outcomes. NIPT, noninvasive prenatal testing; TOP, termination of pregnancy. *Normal phenotype was based on parental reports during follow-up and was not verified by diagnostic karyotyping.

Prenatal diagnosis results showed that 270 of the 377 cases were concordant with NIPT findings, giving an overall PPV of 71.62%. This PPV was calculated based on invasive prenatal diagnosis alone. Stratified by trisomy type, the PPV for T21 was 84.21% (240/285), for T18 was 56.00% (28/50), and for T13 was 4.76% (2/42). The karyotype composition of T21 included 214 cases of 47, XN, + 21, 6 cases of 46, XN, + 21, der (21;21)(q10;q10), 3 cases of 46, XN, der (14;21)(q10;q10), +21, 11 cases of mosaic T21, 1 case of 48, XN, + 21, +mar, 1 twin pregnancy with both fetuses being 47, XN, + 21, and 4 twin pregnancies with one fetus being 47, XN, + 21 and the other 46, XN. For T18, the karyotypes comprised 27 cases of 47, XN, + 18 and 1 case of partial T18 with 46, X, der (18)t(X;18)(q13;q21.3). Notably, among the T13 false-positive cases, 2 were confirmed by CMA to have uniparental disomy (UPD) of chromosome 13. Among the 270 true-positive cases, 262 chose termination of pregnancy, 4 T21 cases continued the pregnancy, and 4 twin pregnancies underwent selective fetal reduction and delivered normal infants.

Of the 104 positive cases that did not undergo prenatal diagnosis, 77 opted for termination due to fetal demise or multiple malformations, and 27 delivered live births. Of the 27 live births, 3 were postnatally confirmed as T21 by peripheral blood karyotyping, while the remaining 24 showed no abnormalities upon follow-up. After incorporating these 3 cases, the corrected PPV for T21 was 84.38% (243/288), and the overall corrected PPV was 71.84% (273/380) (**Table 1**). This corrected PPV, which incorporates the three postnatally confirmed cases in women who declined prenatal diagnosis, is used as the primary result throughout this study.

Further stratified analysis using the corrected PPVs showed that the corrected PPV for T21 was 84.38% (243/288), for T18 was 56.00% (28/50), and for T13 was 4.76% (2/42). The differences in corrected PPVs among the three trisomy types were statistically significant (χ² = 121.99, P< 0.001).

In addition, during subsequent verification, 6 cases of maternal 47, XX, + 21 (5 cases of full 47, XX, + 21 and 1 case of mosaic 47, XX, + 21[4]/46, XX[32]) were identified. Among these, the fetal prenatal diagnosis results were 46, XN in 5 cases and 47, XN, + 21 in 1 case. Of these, the 5 cases with a normal fetal karyotype (46, XN) were classified as false positives and are described in Section 3.4; the remaining case, in which the fetus was confirmed as 47, XN, + 21, was a true positive and was therefore not included in the false-positive mechanistic analysis.

### 3.2 Stratified analysis of maternal age and serum screening characteristics with NIPT PPV

Among the 243 true-positive T21 cases, maternal age ranged from 17 to 47 years (mean 33.14 years). Serum screening data were available for 117 cases, with T21 risk estimates ranging from 1/2 to 1/35, 995. Among the 45 false-positive T21 cases, maternal age ranged from 17 to 43 years (mean 29.40 years), with serum data available for 22 cases (risk range: 1/93 to 1/60, 427).

Among the 28 true-positive T18 cases, maternal age ranged from 21 to 42 years (mean 33.46 years), with serum data available for 12 cases (risk range: 1/5 to 1/6, 328). Among the 22 false-positive T18 cases, maternal age ranged from 23 to 43 years (mean 32.05 years), with serum data available for 8 cases (risk range: 1/727 to 1/10, 000).

Stratified analyses of corrected PPV by maternal age and serum screening risk are summarized in **Table 2**. For T21, advanced maternal age (≥35 years) was associated with a significantly higher PPV compared with younger women (91.06% vs. 79.39%, χ² = 6.41, P = 0.011). For T18, the PPV difference between age groups did not reach statistical significance (63.64% vs. 50.00%, χ² = 0.46, P = 0.498).

**Table 2:** Stratified analysis of corrected PPV by maternal age and serum screening risk.

Trisomy	Stratification factor	Subgroup	NIPT-positive (n)	True-positive (n)	Corrected PPV (%)	χ²/Fisher	P value
T21	Maternal age	≥35 years	123	112	91.06	6.41	0.011
		<35 years	165	131	79.39		
	Serum risk	High (>1/270)	84	80	95.24	20.88^a^	<0.001^a^
		Intermediate (1/270–1/1000)	44	31	70.45		
		Low (<1/1000)	11	6	54.55		
T18	Maternal age	≥35 years	22	14	63.64	0.46	0.498
		<35 years	28	14	50		
	Serum risk	High (>1/350)	9	9	100	-	0.001^b^
		Non-high (≤1/350)	11	3	27.27		

^a^χ² for trend; ^b^Fisher’s exact test (high-risk vs. non-high-risk).

For serum screening risk in T21, PPV decreased progressively from 95.24% in the high-risk group to 70.45% in the intermediate-risk group and 54.55% in the low-risk group, with a significant decreasing trend (χ² for trend = 20.88, P< 0.001). For T18, the PPV in the high-risk group was 100% (9/9), significantly higher than that in the non-high-risk group (27.27%, 3/11; Fisher’s exact test, P = 0.001).

### 3.3 Comparison of NIPT-Z-scores between true-positive and false-positive cases

Among the 45 T21 false-positive cases, 4 were confirmed by maternal peripheral blood karyotyping as 47, XX, + 21, with NIPT-Z-scores of 72.01, 75.98, 79.19, and 80.86, respectively. These Z-scores markedly deviated from those of the other false-positive cases; they were therefore excluded from subsequent analyses. After exclusion, the mean Z-score of the remaining 41 T21 false-positive cases was 5.08 ± 2.04 (**Figure 2A**), which was significantly lower than that of the 243 T21 true-positive cases (17.60 ± 7.34) (P< 0.001). Stratified analysis by Z-score showed that the PPV for T21 progressively increased with higher Z-scores: PPV was 21.95% (9/41) for 3< Z ≤ 6, 81.58% (31/38) for 6< Z ≤ 10, 98.31% (116/118) for 10< Z ≤ 20, and 100% (87/87) for Z > 20 (**Figure 3A**).

**Figure 2:**
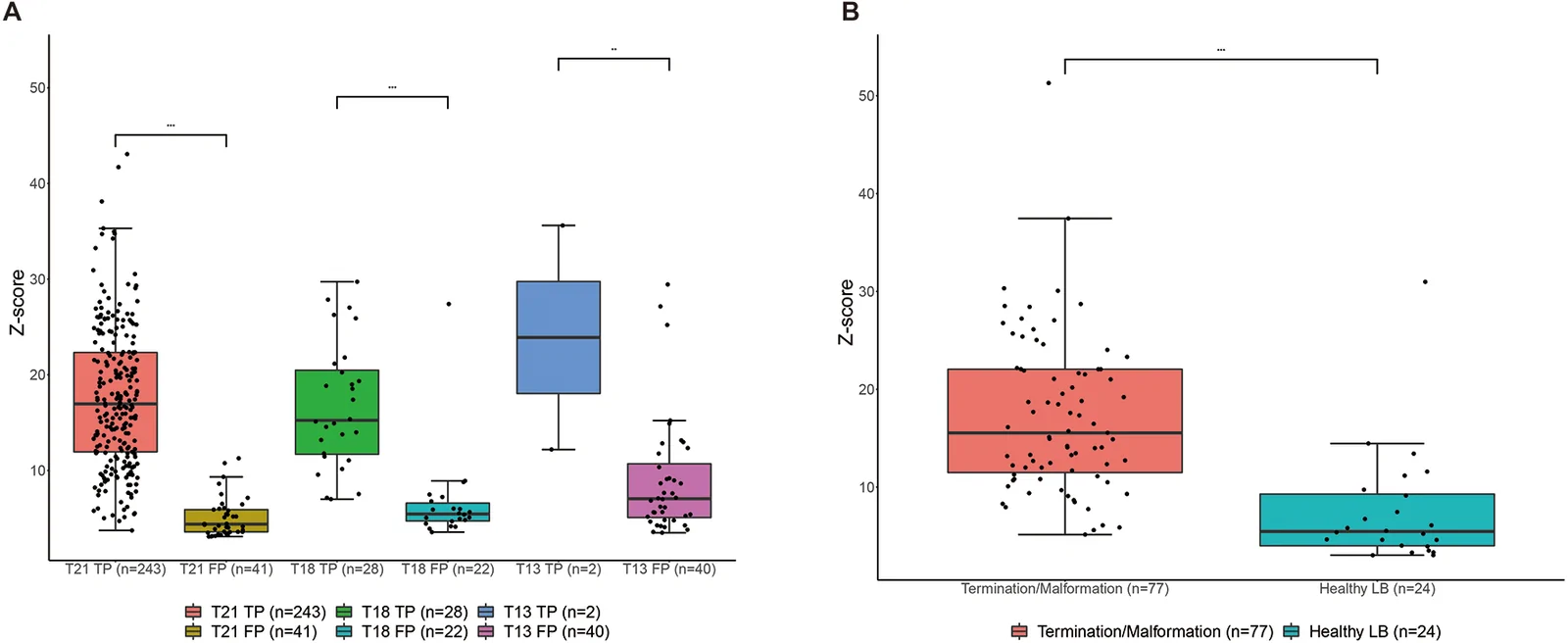
Boxplots of NIPT-Z-score distribution across different groups. **(A)** Comparison of Z-scores between true-positive (TP) and false-positive (FP) cases for T21, T18, and T13. The 4 T21 FP cases attributable to maternal 47, XX, + 21 (Z-scores: 72.01–80.86) were excluded. Group comparisons were performed using independent samples t-tests. **(B)** Comparison of Z-scores between positive cases with adverse pregnancy outcomes (termination due to fetal demise or multiple malformations) and those with healthy live births. TP, true-positive; FP, false-positive; LB, live birth. *P< 0.05; **P< 0.01; ***P< 0.001.

**Figure 3:**
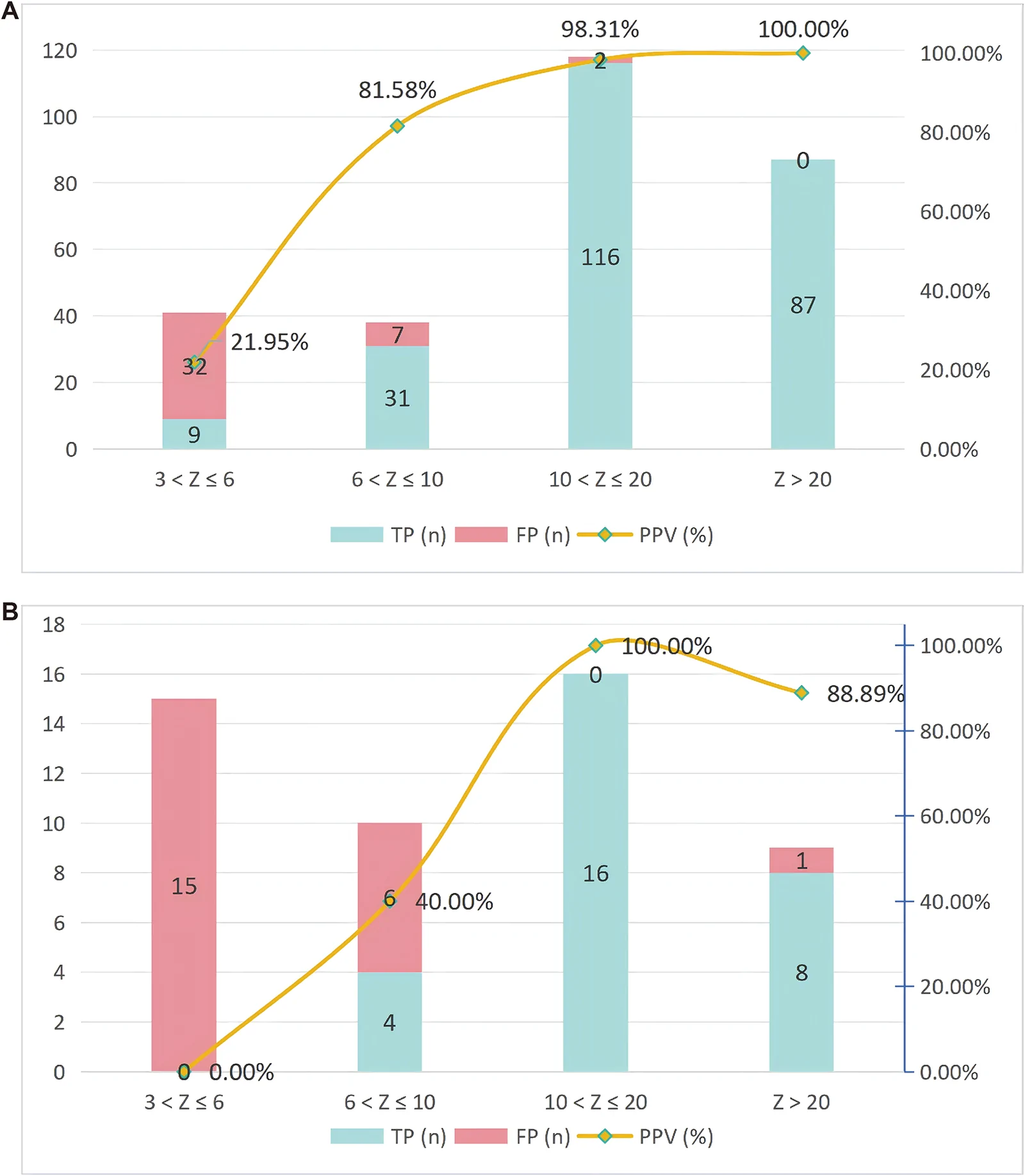
Positive predictive value (PPV) stratified by NIPT-Z-score intervals for T21 and T18. **(A)** For T21, PPV increased progressively with higher Z-score intervals: 21.95% (9/41) for 3< Z ≤ 6, 81.58% (31/38) for 6< Z ≤ 10, 98.31% (116/118) for 10< Z ≤ 20, and 100% (87/87) for Z > 20. **(B)** For T18, PPV was 0% (0/15) for 3< Z ≤ 6, 40% (4/10) for 6< Z ≤ 10, 100% (16/16) for 10< Z ≤ 20, and 88.89% (8/9) for Z > 20. TP, true-positive; FP, false-positive; PPV, positive predictive value.

In T18 cases, the mean Z-score of the 28 true-positive cases was 16.78 ± 6.51, whereas that of the 22 false-positive cases was 6.63 ± 4.86, with a statistically significant difference between the two groups (P< 0.001). Stratified analysis by Z-score showed that PPV was 0% (0/15) for 3< Z ≤ 6, 40% (4/10) for 6< Z ≤ 10, 100% (16/16) for 10< Z ≤ 20, and 88.89% (8/9) for Z > 20 (**Figure 3B**).

In T13 cases, the Z-scores of the 2 true-positive cases were 12.19 and 35.61, respectively, whereas the mean Z-score of the 40 false-positive cases was 9.01 ± 6.20. Due to the limited number of T13 true-positive cases, stratified PPV analysis by Z-score was not performed.

Additionally, among the 101 positive cases that did not undergo diagnostic testing, the NIPT-Z-score of the 77 cases that opted for termination of pregnancy due to fetal demise or multiple malformations was 17.30 ± 8.06, significantly higher than that of the 24 cases that delivered healthy live births (7.54 ± 6.01) (P< 0.001) (**Figure 2B**).

The distribution of NIPT-Z-scores across different groups is shown in **Figure 2A** (T21, T18, and T13 true-positive vs. false-positive cases) and **Figure 2B** (adverse outcomes vs. healthy live births). Notably, the 4 T21 false-positive cases attributable to maternal 47, XX, + 21 with extremely elevated Z-scores (72.01–80.86) were excluded and are not shown in **Figure 2A**.

### 3.4 Biological mechanisms underlying false-positive results

Of the 107 prenatally diagnosed false-positive cases, 17 had sufficient additional data for mechanistic investigation; 3 additional cases without prenatal diagnosis but with follow-up evidence of vanishing twin were also included, yielding a total of 20 cases for analysis in **Table 3**. The remaining false-positive cases lacked additional validation data and could not be mechanistically classified.

**Table 3:** Detailed case-level summary of biological mechanisms underlying false-positive NIPT results.

Mechanism	Case ID	Y-Z-score^a^	Fetal fraction (%)	NIPT-reported trisomy	Z-score	Prenatal diagnosis	Postnatal sex	Key finding
Maternal chromosomal abnormality	18GZM5297	33.02	10.28	T21	79.19	46, XN	male	Maternal 47, XX, + 21
	21GZM8388	-0.39	12.80	T21	75.98	46, XN	female	Maternal 47, XX, + 21
	22GZM1079	1.73	12.80	T21	80.86	46, XN	female	Maternal 47, XX, + 21
	22GZM6100	86.34	28.84	T21	8.62	46, XN	male	Maternal 47, XX, + 21[4]/46, XX[32]
	23GZM3457	65.83	22.08	T21	72.01	46, XN	male	Maternal 47, XX, + 21
Vanishing twin	18GZM5156	12.36	2.51	T21	3.05	Not performed	female	Suspected VT; sex discordance
	18GZN5589	12.58	4.08	T21	3.51	Not performed	female	VT at 8 weeks; sex discordance
	19GZM4393	15.04	4.86	T21	6.00	46, XN	female	VT at 7 weeks; sex discordance
	21GZM1572	64.09	21.70	T21	5.44	46, XN	male	VT at 8 weeks; sex concordance
	22GZN8838	12.60	4.28	T18	4.11	46, XN	female	VT at 8 weeks; sex discordance
	22GZN0716	43.97	14.92	T13	11.59	Not performed	male	VT at 10 weeks; sex concordance
	23GZM9320	14.31	4.86	T21	3.75	46, XN	female	Suspected VT; sex discordance
Uniparental disomy	21GZM3886	1.13	17.73	T13	13.15	46, XN	female	CMA: UPD(13)
	22GZM2879	69.01	23.17	T13	29.45	46, XN	male	CMA: UPD(13)
Confined placental mosaicism	20GZM9069	0.06	18.80	T21	3.56	46, XN	female	Placental 10% T21 mosaicism
	21GZM0604	66.38	22.28	T18	27.40	46, XN	male	Placental T18
	21GZM1824	-0.53	17.02	T13	25.21	46, XN	female	Placental T13
Unclassified (normal placental karyotype)	21GZM0576	-0.12	16.90	T21	4.22	46, XN	female	Normal placental karyotype (46, XX)
	21GZM0961	55.55	19.10	T21	3.25	46, XN	male	Normal placental karyotype (46, XY)
	20GZM8064	-0.43	19.62	T18	5.06	46, XN	female	Normal placental karyotype (46, XX)

^a^Y-Z-scores were used for fetal sex determination and fetal DNA fraction calculation. A Y-Z-score > 3 indicates the presence of Y-chromosome sequences, leading to the prediction of a male fetus. NIPT, non-invasive prenatal testing; VT, vanishing twin; CMA, chromosomal microarray analysis; UPD, uniparental disomy. Cases were included only when additional mechanistic data were available. Of the 20 cases, 17 were prenatally confirmed false positives and 3 had follow-up evidence of vanishing twin. One maternal 47, XX, + 21 case was excluded because the fetus was a true positive (47, XN, + 21).

Among the false-positive cases, 5 cases of maternal chromosomal abnormalities were identified, including 4 cases of full 47, XX, + 21 with markedly elevated NIPT-Z-scores (72.01–80.86), and 1 case of mosaic 47, XX, + 21[4]/46, XX[32] with a Z-score of 8.616 (**Table 3**). Notably, in a subsequent pregnancy of the mosaic case, the fetus was confirmed to have T21 by amniocentesis.

Through in-depth analysis of follow-up data, 7 false-positive cases were identified as attributable to vanishing twin. Among them, 5 cases exhibited sex discordance (NIPT-predicted male fetus but female live birth), all presenting with low fetal DNA fraction and low Z-scores; 3 of these had a documented history of early-pregnancy vanishing twin. In addition, 2 cases with NIPT-predicted male and postnatal male confirmation were also identified as vanishing twin during follow-up.

Furthermore, 2 false-positive cases were confirmed by CMA to have UPD of chromosome 13, with Z-scores of 13.15 and 29.45, respectively.

Placental tissue verification was performed in 6 false-positive cases. Among them, 2 cases with relatively high Z-scores were confirmed to have T18 and T13 in placental tissues. Of the remaining 4 cases with relatively low Z-scores, only 1 was confirmed as low-level mosaic T21 (10%), while the other 3 showed a 46, XN karyotype in placental tissues and remained unclassified.

### 3.5 False-negative case analysis

Among the 97, 262 women with negative NIPT results, 4, 567 were lost to follow-up, 2, 264 underwent termination of pregnancy due to incidental findings or other reasons, and 90, 428 delivered phenotypically normal infants. During follow-up of the NIPT-negative cases, 3 false-negative cases were identified and are detailed in **Table 4**.

**Table 4:** Summary of false-negative NIPT cases.

Case ID	Target chromosome	Fetal number	Z-score (initial NIPT)	Z-score (repeat NIPT)^a^	Postnatal/prenatal karyotype	Placental verification
21GZM2853	18	Twin	0.09	-0.32	Twin A: 47, XX, + 18[24]/46, XX[58] Twin B: 47, XX, + 18[23]/46, XX[82]	Not performed
21GZM2865	21	Singleton	0.83	-1.53	47, XX, + 21	Not performed
21GZM4922	21	Singleton	-0.20	1.571; 1.219^b^	47, XN, + 21	CPM with T21 mosaicism^c^

^a^ Repeat testing was performed on stored plasma samples from the initial collection to exclude laboratory errors. ^b^For Case 21GZM4922, repeat testing was performed twice: once on a re-constructed library from the original plasma, and once on a newly collected sample at 28^+3^ weeks of gestation prior to termination. ^c^Placental T21 mosaic ratios by sampling site: maternal edge 21%, maternal center 30%, maternal intermediate 10%, fetal edge 18%, fetal center (site 1) 19%, fetal center (site 2) 21%, and umbilical cord insertion 96%. NIPT, non-invasive prenatal testing; CPM, confined placental mosaicism.

Case 21GZM2853 was a twin pregnancy with a low-risk NIPT result (initial Z-score: 0.09). Both twins were female and were postnatally confirmed as mosaic T18 by peripheral blood karyotyping.

Case 21GZM2865 was a singleton pregnancy with a low-risk NIPT result (initial Z-score: 0.83). The female infant was postnatally confirmed as T21.

Case 21GZM4922 was a singleton pregnancy with a low-risk NIPT result (initial Z-score: -0.20). Mid-trimester ultrasound revealed multiple fetal malformations, prompting prenatal diagnosis, which confirmed T21. Placental multi-site sampling after termination revealed confined placental mosaicism (CPM) with striking regional variation: the T21 mosaic ratio was 96% at the umbilical cord insertion site, 18%-21% at fetal side sites, and 10%-30% at maternofetal side sites (**Table 4**).

To further exclude potential laboratory errors, the stored plasma samples from all 3 cases were re-tested. For Case 21GZM2853 and Case 21GZM2865, repeat testing yielded Z-scores of -0.32 and -1.53, respectively. For Case 21GZM4922, repeat testing was performed twice: once on a re-constructed library from the original plasma (Z-score = 1.571), and once on a newly collected sample at 28^+3^ weeks of gestation prior to termination (Z-score = 1.219). All repeat results remained below the positive threshold (Z< 3), confirming the initial negative findings.

## 4 Discussion

NIPT has been widely adopted in clinical practice as a critical tool for screening fetal chromosomal abnormalities. However, with the expansion of testing scale and the accumulation of clinical data, the discordance between NIPT results and true fetal karyotypes has become increasingly prominent, representing a key bottleneck limiting further improvement of its clinical utility. Based on a large-sample cohort spanning nearly a decade at Ganzhou Women and Children’s Health Care Hospital, this study systematically analyzed the prenatal diagnostic outcomes of NIPT-positive cases and the follow-up data of NIPT-negative cases. We investigated the causes of NIPT discordance from multiple dimensions, including PPV assessment, Z-score stratification, maternal age and serum screening characteristics, biological mechanisms underlying false-positive results, and false-negative mechanisms—with the aim of providing evidence-based guidance for optimizing screening strategies and genetic counseling.

In this study, the PPVs of the three chromosomal aneuploidies differed significantly, with T21 showing the highest PPV (84.38%), followed by T18 (56.00%) and T13 (4.76%), a trend consistent with studies (11, 12, 15). The underlying reason for this disparity lies in the differential natural courses of the three chromosomal diseases (16). T21 presents with the mildest phenotype, and affected fetuses generally survive to the amniocentesis window (3). In contrast, T13 exhibits the most severe phenotype, frequently resulting in embryonic demise during the first or early second trimester, with T18 falling between the two (17). Currently, the testing window for NIPT in China is 12^+0^ to 22^+6^ weeks of gestation, while amniocentesis for prenatal diagnosis is typically performed between 18 and 22 weeks. In the present study, a substantial number of positive cases, particularly those involving T18 and T13, were clinically terminated prior to reaching the amniocentesis window due to ultrasound findings of multiple fetal malformations or intrauterine demise, and thus were not included in the prenatal diagnostic statistics. This observation directly corroborates the aforementioned differences in natural disease course: T13 and T18 fetuses are less likely to survive to the prenatal diagnostic window, which not only contributes to a lower diagnostic uptake rate but also inflates the denominator of PPV calculations with cases lacking karyotypic confirmation, thereby further lowering the estimated PPVs for T18 and T13.

Advanced maternal age is a well-established risk factor for fetal chromosomal aneuploidies (18), and fetal chromosomal abnormalities are associated with characteristic alterations in the placental hormonal profile (19). Consequently, maternal clinical characteristics, including advanced age and positive serum screening results, serve as important references for NIPT clinical indications (20). Our subgroup analysis further quantified this stratification effect: among NIPT-positive cases for T21 and T18, the PPVs were significantly higher in women with advanced maternal age and those with high-risk serum screening results, compared with their non-advanced-age and non-high-risk counterparts. Notably, a considerable proportion of NIPT-positive cases in the non-advanced-age and low-risk serum screening groups were confirmed as true positives by prenatal diagnosis. This finding suggests that although serum screening is less costly, risk stratification based solely on maternal clinical characteristics entails a systematic risk of missed detection (21), further underscoring the clinical value of adopting NIPT as a first-tier screening strategy.

The NIPT Z-score, as a core quantitative metric for chromosomal copy number variations (22–24), demonstrated excellent risk stratification value in this study. Z-scores were significantly higher in true-positive than in false-positive cases, and among positive cases without prenatal diagnosis, those terminated due to fetal demise or multiple malformations exhibited significantly higher Z-scores than those with healthy live births, further supporting the association between Z-scores and clinical outcomes. Stratified analysis by Z-score revealed a significant dose–response relationship between Z-score and PPV for T21: the PPV reached 100% when Z > 20, whereas it was only 21.95% when Z was in the 3–6 range. A similar trend was observed for T18. These findings provide a quantitative decision-making basis for clinical risk stratification based on Z-scores: when Z-scores fall within a low range, the possibility of false positivity should be thoroughly communicated to the pregnant woman, with a recommendation for confirmatory prenatal diagnosis; conversely, when Z-scores are markedly elevated, the likelihood of true positivity increases substantially, though comprehensive interpretation should still incorporate ultrasound findings and other clinical information.

CPM refers to the phenomenon of karyotypic discordance between the placenta and the fetus and is recognized as a major biological basis for both false-positive and false-negative NIPT results (14, 25). Since the cell-free DNA detected by NIPT is primarily derived from apoptotic placental trophoblast cells, when the placenta harbors mosaicism, the cffDNA signals in maternal blood may not accurately reflect the true fetal karyotype (26). CPM was directly verified in a false-negative case (Case 21GZM4922). Placental multi-site sampling revealed a striking gradient of T21 mosaicism: 96% at the umbilical cord insertion, 18%–21% on the fetal side, and 10%-30% on the maternofetal side. Given that maternal circulating cffDNA is primarily derived from trophoblast cells at the maternofetal interface (27), the low-grade mosaicism (10%-30%) in this region likely resulted in a T21 signal below the NIPT detection threshold, leading to the false-negative result. This striking gradient in mosaic distribution provides a direct mechanistic explanation for CPM-induced false-negative NIPT results.

In contrast, the actual contribution of CPM to false-positive cases is more complex (28). Generally, CPM predominantly arises from the mechanism of trisomic rescue (29), in which a trisomic conceptus undergoes loss of one chromosome during development, restoring a normal karyotype in the fetal lineage (30). Depending on the parental origin of the retained chromosomes, trisomic rescue can lead to three distinct genetic outcomes: isodisomy (isoUPD), heterodisomy (heteroUPD), or restoration of normal biparental disomy (31). In this study, two T13 false-positive cases were confirmed by CMA to have isodisomy of chromosome 13 (isoUPD (13)). Although placental histological verification was not performed in these two cases, the presence of UPD itself constitutes indirect evidence of CPM. We hypothesize that trisomic rescue led to fetal isoUPD (13), while the placenta retained the T13 karyotype, resulting in a positive NIPT result despite a normal fetal karyotype.

On the other hand, among the six false-positive cases that underwent placental tissue verification, two cases with relatively high Z-scores were identified as T18 and T13, respectively, with corresponding negative CMA results in the fetuses. These two CMA-negative cases may represent heteroUPD or normal biparental disomy, and the presence of placental T18/T13 directly points to CPM. In contrast, among the remaining four cases with relatively low Z-scores, only one was confirmed as a low-level T21 mosaic (10%), while the other three showed a 46, XN karyotype in placental tissues. Based on these observations, the latter three low-Z-score false-positive cases with normal placental karyotypes are more likely attributable to random data fluctuations or statistical false positivity due to low fetal DNA fraction, rather than to biological factors such as placental mosaicism. Taken together, CPM may account for only a proportion of NIPT false-positive results, with other factors, including maternal chromosomal abnormalities and vanishing twin, also playing substantial roles.

The cell-free DNA detected by NIPT is predominantly derived from the maternal compartment (accounting for approximately 90%–95%), and the analytical algorithms are constructed based on the assumption of a normal maternal karyotype (32, 33). When the mother herself harbors a chromosomal abnormality, this assumption is violated, leading to distorted NIPT results. In this study, four false-positive cases were identified as having maternal full-type 47, XX, + 21, all presenting with markedly elevated NIPT-Z-scores (72.01–80.86). This finding suggests that in NIPT-positive cases with extremely high Z-scores (>70), maternal chromosomal abnormalities should be highly suspected, and maternal peripheral blood karyotyping should be prioritized to rule out interference. In contrast, one case of maternal mosaic 47, XX, + 21 (mosaic ratio approximately 11%) was more deceptive: the Z-score was only 8.616, falling within the range typically observed in true-positive cases. In a subsequent pregnancy of this patient, the fetus was confirmed by amniocentesis to have full trisomy 21, suggesting that low-level maternal mosaicism not only interferes with the interpretation of NIPT results but may also increase the risk of conceiving a fetus with full trisomy 21.

The identification of vanishing twin typically relies on ultrasound findings and requires the patient to proactively report relevant history prior to testing (34). However, in clinical practice, a considerable proportion of pregnant women do not provide such information, and some may not even be aware of the occurrence of vanishing twin. In China, non-medical-need fetal sex determination is strictly regulated, and neither routine NIPT reports nor ultrasound examinations disclose fetal sex, further complicating the identification of occult vanishing twin through conventional clinical pathways. By performing retrospective sex prediction through in-depth mining of raw NIPT data and comparing it with postnatal sex, we successfully identified seven false-positive cases definitively or highly suspected to be attributable to vanishing twin. Among them, five cases showed NIPT-predicted male fetus (detection of Y chromosome) but delivered female infants, and we hypothesize that the vanished fetuses were male embryos with chromosomal abnormalities. Because the NIPT algorithm preferentially employs Y-chromosome reads for fetal DNA fraction calculation when Y chromosome is detected, the residual Y-chromosome DNA misled the algorithm, resulting in sex misclassification and a falsely low measured fetal DNA fraction. Low cffDNA concentration and low Z-scores have been previously recognized as important technical causes of false positivity (22, 35). Our observations suggest that some low-Z-score false-positive cases may actually be attributable to such occult vanishing twin rather than genuinely low fetal DNA fraction. Among the above five cases, three had a documented history of early-pregnancy vanishing twin. Two additional false-positive cases with NIPT-predicted male and postnatal confirmation of male were also identified as vanishing twin during follow-up. These findings indicate that sex-based comparison can effectively detect occult vanishing twin events that are easily missed in routine clinical follow-up. Moreover, identifying vanishing twin through sex discordance carries a certain underdetection rate: when the sex of the vanished fetus matches that of the surviving fetus, sex comparison alone cannot detect the event. Therefore, vanishing twin constitutes an important cause of NIPT false positivity, and its contribution to false positivity may have been significantly underestimated in previous studies (36–38).

The Z-score distribution characteristics of false-positive cases attributable to different etiologies also help explain the observed dose–response relationship between Z-score and PPV. Specifically, false-positive cases caused by maternal full-type chromosomal abnormalities (e.g., 47, XX, + 21) exhibit extremely high Z-scores (>70), though such cases represent a small proportion of false positives. False positives attributable to low-level maternal mosaicism or CPM from trisomic rescue present Z-scores comparable to those of true positives, also constituting a minority. In contrast, false positives resulting from low-level mosaic CPM, vanishing twin, and random data fluctuations or statistical false positivity due to low fetal DNA fraction predominantly manifest as mildly elevated Z-scores, constituting the majority of false-positive cases. This distribution pattern precisely explains why the PPV in the low Z-score range is significantly lower than that in the high Z-score range: the low Z-score range contains a substantial aggregation of mildly elevated false positives (statistical fluctuation, low concentration, vanishing twin, etc.), whereas truly clinically significant high-Z-score signals are more likely to point toward true positivity (39).

Several limitations of this study should be acknowledged. First, this was a single-center retrospective analysis, and the follow-up of NIPT-negative cases relied primarily on routine clinical data; karyotyping was not performed for negative cases, and false-negative cases, particularly those with low-level mosaicism, may have been underestimated. Second, only two T13 true-positive cases were identified, precluding Z-score stratification and subgroup analyses, and thus the generalizability of related conclusions should be interpreted with caution. Third, placental multi-site sampling or maternal in-depth validation was not performed for some false-negative cases, limiting the completeness of false-negative mechanistic analyses. Fourth, the mechanistic analysis of false-positive cases was limited to 20 cases with additional validation data, of which only six had placental verification; this may introduce selection bias, and the true proportion of each mechanism among all false-positive cases remains uncertain. In particular, the contribution of vanishing twin may be underestimated because same-sex twin demise and unreported cases cannot be detected through sex-discordance analysis alone. Future studies should expand the scope of validation.

## 5 Conclusions

In summary, our decade-long experience with nearly 100, 000 cases demonstrates that the causes of NIPT and fetal karyotype discordance exhibit a clear directional divergence. False-positive results are etiologically heterogeneous, encompassing biological factors (CPM, maternal chromosomal abnormalities, and vanishing twin, with the latter being severely underestimated) as well as technical factors (low cffDNA concentration leading to reduced Z-scores, and data-driven statistical fluctuations). In contrast, false-negative results are predominantly attributable to CPM, with an extremely low incidence of 0.003% in this cohort. In such cases, low-proportion or regionally distributed placental mosaicism results in abnormal signals falling below the NIPT detection threshold, leading to missed detection. Through systematic analysis of clinical data, Z-score stratification, and follow-up information, the majority of discordance causes can be effectively identified, providing precise evidence for clinical genetic counseling and pregnancy management. The analytical framework and experience established in this study may serve as a practical reference for optimizing NIPT screening strategies and reducing unnecessary invasive prenatal diagnostic procedures in other regions.

## Data Availability

The original contributions presented in the study are included in the article/**Supplementary Material**. Further inquiries can be directed to the corresponding authors.
